# Combining Cryo-EM Density Map and Residue Contact for Protein Secondary Structure Topologies

**DOI:** 10.3390/molecules26227049

**Published:** 2021-11-22

**Authors:** Maytha Alshammari, Jing He

**Affiliations:** Department of Computer Science, Old Dominion University, Norfolk, VA 23529, USA; malsh009@odu.edu

**Keywords:** protein structure, cryo-electron microscopy, secondary structure, contact, amino acid, topology, image, constraints

## Abstract

Although atomic structures have been determined directly from cryo-EM density maps with high resolutions, current structure determination methods for medium resolution (5 to 10 Å) cryo-EM maps are limited by the availability of structure templates. Secondary structure traces are lines detected from a cryo-EM density map for α-helices and β-strands of a protein. A topology of secondary structures defines the mapping between a set of sequence segments and a set of traces of secondary structures in three-dimensional space. In order to enhance accuracy in ranking secondary structure topologies, we explored a method that combines three sources of information: a set of sequence segments in 1D, a set of amino acid contact pairs in 2D, and a set of traces in 3D at the secondary structure level. A test of fourteen cases shows that the accuracy of predicted secondary structures is critical for deriving topologies. The use of significant long-range contact pairs is most effective at enriching the rank of the maximum-match topology for proteins with a large number of secondary structures, if the secondary structure prediction is fairly accurate. It was observed that the enrichment depends on the quality of initial topology candidates in this approach. We provide detailed analysis in various cases to show the potential and challenge when combining three sources of information.

## 1. Introduction

Cryo-electron microscopy (cryo-EM) is a biophysical technique for determination of molecular structures. Over the last ten years, many atomic structures of molecules have been successfully derived using this technique, such as for viruses [1,2], proteasomes [3], and membrane-bound proteins [4,5]. As of October 2021, there are 6977 atomic structures in the Protein Data Bank (PDB), for which electron density maps with better than 5 Å resolution were obtained using the cryo-EM technique. For density maps with better than 5 Å resolution, the backbone of a protein chain is often distinguishable, and near-atomic structures can be derived, although a high-accuracy structure often requires a density map with a resolution near 3 Å. For a density map with lower than 5 Å resolution, it is challenging to derive the atomic structure from the density map directly, since molecular details are less resolved. As of October 2021, there are 1221 atomic structures derived from density maps with medium resolution (5–10 Å). Note that there is a much smaller number of atomic structures derived from medium-resolution density maps than structures from maps with better than 5 Å resolution. Since molecular details are not sufficient to determine atomic structures for most medium-resolution density maps, template-based methods are mainly used to derive atomic structures from such maps. A template is an atomic structure that shares sufficient structure similarity with the target protein. Fitting of a template into the density map is performed to model the entire length of the target protein [6,7,8,9,10,11].

When no suitable template structures are available, such as for a new fold, matching secondary structures that are detected from the density map with those predicted from the sequence of the protein is a promising method to derive the arrangement of secondary structures in 3-dimensional space (3D) [12,13,14,15,16,17,18,19]. The relative positioning of secondary structures in 3D provides critical information to derive the tertiary structure of a protein, since secondary structures are often major components of a tertiary structure.

**Figure 1:**
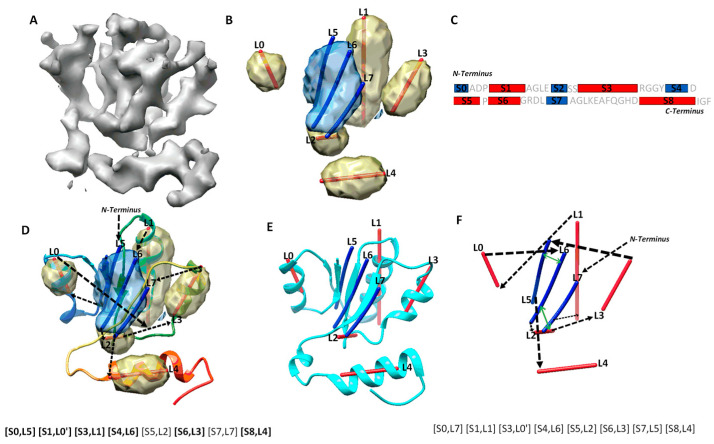
Secondary structure sequence segments, image traces, topology, and pair-contact. (**A**) The cryo-EM density map (gray, EMDB ID 6810) component that corresponds to chain H of atomic structure 5y5x (PDB ID). (**B**) The detected secondary structure regions of α-helices (yellow density) and β-sheet (blue density) using DeepSSETracer [20]. α-traces (red lines) were derived using Principle Component Analysis for α-helices, and β-traces (blue lines) were predicted for β-strands using StrandTwister [21]. (**C**) An illustration of the amino acid sequence of protein 5y5x chain H annotated with the locations of helices (red rectangles) and β-strands (blue rectangles) predicted using JPred [30]. (**D**) An example of a correct topology shown as a diagram and as a list of mapped pairs. Black arrows indicate the topology, with the order of the secondary structure traces from N to C terminal and direction of each trace. The atomic structure of 5y5x (PDB ID) chain H is shown as a rainbow ribbon. The correctly mapped secondary structure pairs are highlighted in the representation of the topology. (**E**) The atomic structure (cyan ribbon) of chain H in 6r0z superimposed with the secondary structure traces. (**F**) An example of a wrong topology indicated using black arrows. The secondary structure contact pairs derived from significant long-range amino acid contacts are indicated as green arrows for the wrong topology.

Protein secondary structures, such as α-helices and β-sheets, are the most distinguishable characteristics in a medium-resolution cryo-EM density map, even though amino acids are not discernible at such a resolution. In most medium-resolution maps, an α-helix resembles a cylinder, and a β-sheet appears as a thin layer of density in a medium-resolution map, although the general shape characters may be affected by their sizes and the density from local environment of the molecule. As an example, five helix regions and one β-sheet region were identified using DeepSSETracer, a secondary structure detection method built on convolutional neural networks (CNN) [20] (Figure 1B). A segmented helix region is represented by the central line (also referred as α-trace) using Principle Component Analysis. A segmented β-sheet region can be represented using a set of lines (also referred as β-traces) for β-strands using StrandTwister [21]. StrandTwister utilizes the twist of a β-sheet to derive possible orientations of β-strands from a segmented β-sheet region. In principle, it is possible to use a set of lines to represent the orientation and position of major helices and β-strands in the cryo-EM density map of them medium resolution.Various methods have been developed to detect secondary structure elements such as α-helices and β-sheets from cryo-EM density maps [22,23,24,25,26,27,28,29]. In practice, accurate detection of secondary structure is challenging, since the detection may miss or wrongly detect a helix/β-strand. As an example, eight secondary structure traces were detected, labeled from L0 to L7, from the cryo-EM density map (Figure 1B,D). Four of the five helices are correctly detected, since they are in the proximity of the helices in the atomic structure. L2 was a wrongly detected small helix region at a turn of the atomic structure. Three β-strands in the β-sheet (blue) were detected, and two of them, L5, and L6, are close to the two strands in the atomic structure (Figure 1D). The third detected β-trace, L7, corresponds to a loop in the atomic structure 5y5x (PDB ID). In general, secondary structure traces show relative geometric relationship among secondary structures, although such information needs to be linked with the sequence of amino acids to derive the tertiary structure of a protein.

Protein secondary structure prediction is a well-studied problem, and many methods exist to predict segments of a protein sequence for secondary structures [31,32]. As an example, five helices and four β-strands were predicted from an amino acid sequence using JPred [30] (Figure 1C). Four of the five helix segments and three of the four β-strand segments are predicted correctly, since they co-locate approximately with the secondary structures of the atomic structure. Although most secondary structures can be predicted from the sequence and the density map. Some are inaccurate, with undetected and/or wrongly detect secondary structures. There is a need to combine two sources of information, one from the sequence and the other from the 3D image, to maximize the knowledge about secondary structures.

Mapping secondary structure traces predicted from a cryo-EM 3D image to segments of amino acid sequence is referred to as the process of finding the topology of secondary structures [15,16,17]. The problem of finding the optimal secondary structure topology is an NP-hard problem [13], although the number of secondary structures is bounded. Let us use a simplified example, in which only helices are in the structure, in order to consider the nature of the problem. Suppose *N* α-traces are detected from a cryo-EM density map, and *M* helix segments are predicted from the protein sequence, *M ≥ N*. A topology of helices describes the order of the *N* helix traces and the direction of each trace with respect to the direction of the protein sequence. In total, there are $\left(\begin{array}{c} M\\ N \end{array}\right)N!2^{N}$ topologies for helices. This is because there are $\left(\begin{array}{c} M\\ N \end{array}\right)$ ways to pick *N* from *M* secondary structures, and for each set of *N* traces, there are N! different orders and two possible directions for each trace. For a protein with both helices and β-strands, sequence segments and traces are matched separately for helices and β-strands, respectively. For an example, in the topology in Figure 1D, the order of the eight detected secondary structure traces is (L5, L0, L1, L6, L2, L3, L7, L4) starting from N-terminal to C-terminal of the sequence. Suppose we use L5 and L5′ to distinguish two possible directions to align trace L5 with the protein sequence and do similarly for other traces. The above-mentioned topology is represented as a list ([S0, L5], [S1, L0′], [S3, L1], [S4, L6], [S5, L2], [S6, L3], [S7, L7], [S8, L4]), in which the order is reflected by matching each trace to a specific sequence segment from N to C terminal. In this topology, all six correctly detected secondary structure traces are mapped to sequence segments at approximately correct locations on the sequence, represented as ([S0, L5], [S1, L0′], [S3, L1], [S4, L6], [S6, L3], [S8, L4]) (highlighted in Figure 1D). An example of an incorrect topology is shown in Figure 1F, with an incorrect order of the six correctly detected traces, as in (L1, L0, L6, L3, L5, L4).

Existing methods for deriving topologies are based on matching the geometric information of secondary structure traces with that of the predicted sequence segments [12,13,15,16,17]. The geometrical information includes the length of a secondary structure and the distance between two consecutive secondary structures. Abeysinghe et al. use a graph-matching algorithm using A* search to relate two graphs, one created from secondary structure traces and the other from sequence segments [12]. Al Nasr et al. use a dynamic programming method to find the top-ranked possible topologies in one graph [13,15]. Biswas et al. [16,17] expand the approach of Al Nasr et al. [15] to employ a dynamic programming method, MultiTopoDP, to include multiple sets of secondary structure predictions from the protein sequence. This shows that the topology accuracy is improved when secondary structure predictions from multiple servers are considered, since different servers may predict well for different secondary structures. Although the algorithmic advantage was demonstrated in MultiTopoDP, the application of the method was limited by the requirement of *M ≥ N*. In this paper, this requirement is eliminated, and the method can be generally applied to all test cases, including those with more predicted secondary structure traces than segments (*M < N*). The method in this work allows us to investigate the topology problem in broader cases. A test set contains 14 cases with a variety of types of cases—α-proteins (9), α + β proteins (5), experimentally derived cryo-EM map components (6), simulated density maps (8), CAPS targets (3), small proteins with less than 150 amino acids (6), and larger proteins with length between 150 and 345 (8). Results from this diverse set of test cases allow us to understand both the potential and challenge in the problem of secondary structure topology when using multiple sources of information.

Prediction of amino acid contact pairs from a protein sequence has been shown as a critical step in prediction of tertiary structures [33,34,35]. The accuracy of contact pairs has enhanced significantly over the last ten years, as demonstrated by recent events in Critical Assessment of Structure Proteins (CASP) [36,37,38,39,40,41,42]. For ab initio approaches, it is possible to predict tertiary structures with good accuracy [43,44,45]. For example, AlphaFold2 achieved a median score of 87.0 Global Distance Test (GDT) in the free modeling category [43]. However, it is challenging to predict accurately for large proteins, particularly those with complicated relationships of secondary structures. The geometric information about secondary structures derived from cryo-EM density maps provides complementary information to the contact pair information. How to combine them is an interesting question, since each describes the information at a different level, and none are perfect. In this paper, we propose a method to map amino acid pairs to secondary structure traces, so that the geometric relationship can be combined with predicted contact pairs to enrich the topological predictions for secondary structures. We previously performed a pilot study to involve contact information in topology prediction of secondary structures [46]. In this paper, we describe a substantially more extensive study and show different effects observed when using contact pairs in four categories of cases. The design of our approach is to extract the most significant long-range contact pairs and map them to secondary structure traces, for which shortest distance was evaluated for satisfactory contact. Results from our approach show that the use of significant long-range pairs is most effective for large proteins or proteins with complicated relationships of secondary structures. The topology prediction of secondary structures is enriched for cases in this category of cases, while it is not in small proteins where secondary structure prediction is fairly accurate from both the cryo-EM maps and the sequence. When the secondary structure prediction is fairly wrong, either from the cryo-EM map or the sequence, the use of amino acid contact pair information may hurt the topology prediction.

## 2. Results

### 2.1. Evaluation of Topologies of Secondary Structures

Possible secondary structure topologies are derived from an optimization of agreement between a set of predicted traces from a cryo-EM map and a list of predicted sequence segments for secondary structures. In an ideal case, in which all secondary structures in the atomic structure are predicted, both from the protein sequence and from the cryo-EM map, different possible topologies differ only in the order and direction of secondary structure traces. However, when there are secondary structures missed and/or wrongly detected, only a subset of secondary structures could be directly evaluated for matches between sequence segments and traces when compared with the atomic structure. We define the maximum-match topology to be the one with the maximum number of correctly matched pairs, each consisting of a predicted secondary structure sequence segment and a trace. For correctly matched pairs, the order of secondary structure traces and the direction of each follow the direction of the sequence from N to C terminal. As an example, the maximum-match topology produced for a cryo-EM component map of 6810 (EMDB ID) is ([S0, L5], [S1, L0′], [S3, L1], [S4, L6], [S5, L2], [S6, L3], [S7, L7], [S8, L4]), since it has the maximum number of six pairs of correctly matched secondary structures ([S0, L5], [S1, L0′], [S3, L1], [S4, L6], [S6, L3], [S8, L4]) (highlighted in Figure 1D). Note that [S5, L2] and [S7, L7] are not counted towards the maximum number, since S5, S7, L2, and L7 are not correctly predicted secondary structures according to 5y5x (PDB ID), even though the two pairs matched approximately correctly. Since there may be multiple maximum-match topologies using different subsets of secondary structures, the highest rank of the maximum-match topology and the number of correctly matched secondary structure pairs were used and reported in evaluation of the performance.

### 2.2. A Case Study for Secondary Structure Prediction and Evaluation of Topologies

The accuracy of secondary structure prediction, either from the protein sequence or from the cryo-EM density map, plays an important role in identification of a correct topology. Let us take a look at one of the cases, in which the secondary structures are predicted well both from the protein sequence and from the cryo-EM component density map. We report how the performance was analyzed and how multiple atomic structures of the same sequence are considered when available. The atomic structure of chain H of 5y5x (PDB ID) contains five helices and one β-sheet. Two of the helices, annotated using STRIDE, are consecutive short helices at the region of L0 α-trace (Figure 1D). JPred predicted five helices, four of which (S1, S3, S6, S8) are approximately in the correct location and one of which (S5) is wrongly predicted at a turn region. It also predicted four β-strands, three of them are approximately at the correct location of the sequence. DeepSSETracer detected five helix regions (yellow), four (L0, L1, L3, L4) approximately at the correct locations and one (L2) at the turn region (interestingly, at a similar region to the one predicted wrongly by JPred) (Figure 1D). The fact that both tools made a similar mistake reflects the challenge in distinguishing a turn from a short helix either on a protein sequence or in cryo-EM density. Four segmented helix regions were represented with four lines using Principle Component Analysis. The lines appear to align well with helices, approximating the central axes of the helices. DeepSSETracer correctly detected the only β-sheet region of the atomic structure (blue) (Figure 1B,D). The β-sheet of 5y5x chain H contains three β-strands, and two of them are in the vicinity of two β-traces (L5, L6) derived using StrandTwister (blue lines in Figure 1D). The orientation of the three β-traces aligns well with two of the three β-strands and a long loop at the β-sheet region (Figure 1D). Even though the long loop was not annotated as a β-strand, it is in proximity to the neighboring β-strand. Our investigation found that there are two alternative structural annotations of the same sequence—chain H of 5y5x and chain H of 6r0z. Alignment of the two chain structures, chain H of 5y5x and 6r0z, was performed using the MatchMaker function in Chimera [47]. We observed that the detected β-traces align better with chain H of 6r0z, since β-trace L7 region is annotated as a β-strand in 6r0z, but as a loop in 5y5x (Figure 1E). The detection of secondary structure traces was based on the 5 Å resolution density map EMD-6810, from which 5y5x atomic structure was derived. The atomic structure of 6r0z was derived from the density map EMD-4702, which has 3.8 Å resolution. The minor difference between the two structures of the same sequence may reflect the flexibility of a protein or the resolution difference of density maps. Nevertheless, utilizing both structures provides better understanding of the quality of predicted secondary structures. In this case, if both 5y5x and 6r0z are considered for the atomic structure of chain H, the overall prediction of secondary structures is good, since four of the five helices and three of the four β-strands are detected at approximately the correct positions. Although minor mistakes exist for a short helix and a β-strand, the correct topology is still ranked first, possibly because secondary structures are well predicted from both the sequence and the density map. The initial topologies were calculated using MultiTopoDP, a graph algorithm to rank possible topologies using overall agreement between loop lengths predicted from the sequence and the distance between secondary structures measured along the skeleton of the 3D image [17]. Details of the dynamic programming algorithm used in MultiTopoDP can be found in Al Nasr et al. [15]. The top-ranked topology is represented as a list of matched pairs ([S0, L5], [S1, L0′], [S3, L1], [S4, L6], [S5, L2], [S6, L3], [S7, L7], [S8, L4]). Since four β-strand segments were predicted and only three β-traces were detected, only three are matchable and are shown in each topology candidate. For example, in the top-ranked topology, β-strand segment S2 is not selected, while the rest three β-segments are. If 5y5x is used as the reference atomic structure, six pairs of secondary structures were correctly matched and can be represented as ([S0, L5], [S1, L0′], [S3, L1], [S4, L6], [S6, L3], [S8, L4]). If 6r0z is used as the reference atomic structure, seven pairs are correctly matched, with an addition of pair [S7, L7]. In general, a greater number of correctly matched secondary structure pairs in a topology suggests closer representation of the atomic structure in terms of secondary structure relationship.

### 2.3. Secondary Structure Contact Pairs and Their Effect in Ranking Initial Topologies

Amino acid contact pairs were produced using either MULTICOM [40,41] or RaptorX [42]. Significant long-range pairs were extracted and were mapped to predicted secondary structure segments. The significance of a pair of amino acids was evaluated based on its p-value (details in Methods). The study in this paper is designed to utilize the most significant set of contacts that are most reliable. All significant long-range amino acid contact pairs are correctly predicted, based on examination against the atomic structures (the numbers shown in Table 1). For 6810-5y5x-H, five pairs of long-range amino acid contacts have p-values higher than three standard deviations (3SD), and four of them are mapped to a pair of sequence segments (S4, S7) that are two neighboring β-strands in the atomic structure. In this case, 85 pairs of significant long-range residue contacts were extracted, and 5 of the 85 pairs involve two secondary structures that were predicted using JPred (Table 1) [30]. This suggests that although many more pairs have significant p-values, most of them involve at least one non-secondary structure region, such as a turn or a loop. When significant long-range residue pairs are mapped to secondary structure segments predicted using JPred, correct residue pairs may be mapped to wrongly predicted secondary structures, as we observed in case T1031 (details later in the section), although we observed that most of the secondary structure contacts (Table 1) derived are correct when they are examined with the atomic structure. For example, four of the five pairs of secondary structure contacts in case 1HG5 are correct pairs ((S1, S3), (S1, S2), (S2, S3), (S3, S4)), but pair (S7, S9) is wrong, since S9 is a wrongly predicted helix on the sequence (details later in the section).

One of the purposes of this study is to investigate the effectiveness of amino acid contact pair information for topology determination. The aim is to enrich the ranking of those initial topologies that satisfy the contact distances at the secondary structure level. The assumption is that when two amino acids are in contact, those belonging to two secondary structures require the pair of secondary structures to be close. Since each initial topology matches predicted sequence segments to traces, the pair of traces with the pair of amino acids could be evaluated for their distance in three-dimensional space. Those topologies with more pairs of secondary structure pairs satisfying distance constraints are re-ranked higher. A re-ranking score (see Section 3.5) was used to consider both the initial topology score and the satisfactory of contact pairs, so that it is less affected by the error from either side of the evaluation.

**Figure 2:**
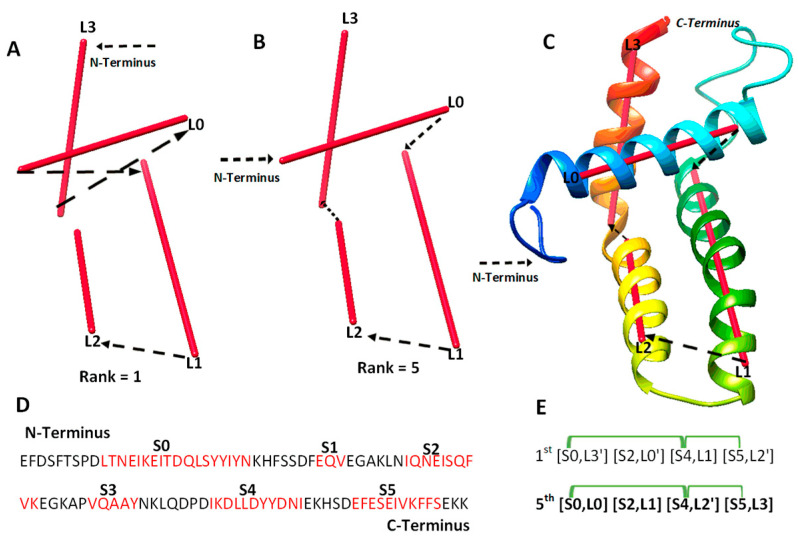
The maximum-match topology for case T1033. (**A**) The 1st ranked topology indicated with black arrows from the N to C terminus of the protein. The α-traces detected from the simulated density map are shown in red. (**B**) The 5th ranked topology is the maximum-match topology, similarly shown as in (**A**). (**C**) The atomic structure of T1033 (rainbow ribbon) superimposed with the 5th topology. (**D**) The amino acid sequence of protein T1033 and helices (red) predicted using JPred [30]. (**E**) Representation of the 1st and the 5th ranked topology. Correctly matched secondary structure pairs are highlighted in the maximum-match topology. Secondary structure contact pairs are marked in green for satisfaction of the distance requirement of 13 Å.

We investigated 14 cases with sequence length ranging from 95 to 345 amino acids. Nine of the cases are α-proteins, and five are α + β proteins. For six of the cases, cryo-EM density maps were extracted from EMDB for the component chains, and simulated density maps were generated for the remaining eight cases using the atomic models and Chimera. Three of the cases are CASP targets in the ab initio category. Results from the 14 test cases show both the potential of deriving the correct topology in a small set of candidates and the challenges to overcome. Four categories of effect were observed using amino acid contact pairs. In the first category involving five cases (6810-5y5x-H, 9534-5gpn-Ae, 3948-6esg-B, 8357-5t4o-L, T1033), the maximum-match topology was ranked high, between the 1st and the 5th in the initial topology list, after MultiTopoDP was applied to the predicted sequence segments and the image traces (Table 2). The use of significant long-range contact pairs did not enhance the ranking of the maximum-match topology for those five cases. We observed that secondary structures are well-detected from both the sequence and the image. Even though they are not perfectly detected, they appear to be best detected when compared to other test cases. Those five cases are also smaller and have less complicated secondary structure relationships when compared to some cases in other categories. In fact, the length of the five cases ranges from 100 to 177 amino acids. It is possible that the significant long-range contact pairs provide limited enrichment when the maximum-match topology is already ranked high in initial topologies. In an analysis of case T1033, a small protein with four helices, significant secondary structure contact pairs do not distinguish well among initially top-ranked topologies, since all four helices are close to each other (Figure 2). More detailed representation of contacts is needed in order to enrich topology ranking for small proteins.

**Figure 3:**
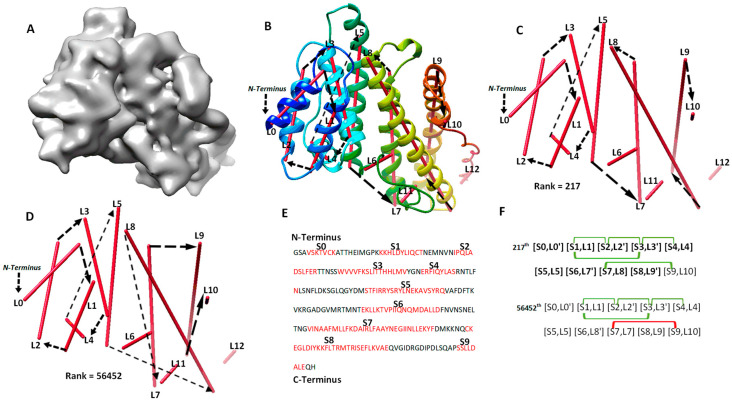
The maximum-match topology for case 1HG5 (PDB ID) after significant long-range contact pairs are applied. (**A**) The simulated density map of chain A of 1HG5 (PDB ID). (**B**) The 217th topology superimposed with the atomic structure (rainbow ribbon). The direction of each α-trace (red line) and the order of α-traces in the topology are indicated with black arrows. (**C**) A separate view of the 217th ranked topology, the maximum-match topology. (**D**) The 56452th ranked topology. (**E**) Secondary structures predicted using JPred [30], with helices annotated in red. (**F**) Representation of the 217th and the 56452th topology. The correctly matched pairs in the maximum-match topology are highlighted. Secondary structure contact pairs are marked in green and red for satisfaction and dissatisfaction of the distance requirement of 13 Å, respectively.

The second category involves four cases (8518-5u8s-A, 1HG5, 3ACW, T1029), for which significant long-range contact pairs enhance the ranking of the maximum-match topology from the initial topology list. These four cases contain complicated secondary structure relationships. Three of the four cases are between 208 and 293 amino acids long, and all four cases have 8 to 15 secondary structures. When the secondary structures are not precisely detected in a large or complicated protein, it is challenging to rank the maximum-match topology high on the list if only the sequence segments and image traces are used. We observe that the use of significant long-range contact pairs enhances the rank significantly. As an example, the maximum-match topology rank was enhanced from the 1022nd to the 217th for 1HG5 (Table 2). For this case, the maximum-match topology includes the correct order and direction for 9 of the 11 secondary structures in the protein (column 3 and 6 in Table 2). In total, 10 of the 13 α-traces detected using DeepSSETracer are correct. L6, L11, and L12 are wrongly detected traces, and they were not selected in the 217th topology (Figure 3). L10 is correctly detected at a small helix with one turn (Figure 3B,C) but is matched to S9, which is a wrongly predicted helix on the sequence. Since [S9, L10] is near the C-terminal end of the sequence and L10 is extremely short, the order and direction of all the 10 traces are correct, although only nine correct matches (highlighted in Figure 3F) are considered in the maximum-match topology. Since the maximum-match topology satisfies all the five pairs of secondary structure contact, it was enriched from the 1022nd to 217th in rank (Table 1 and Figure 3F). We also observed that many other topologies satisfy the five pairs of contact. For a large protein, there needs to be more comprehensive representation of contacts at the secondary structure level for further enrichment.

The third category involves four cases (2620-4uje-BH, 3LTJ, 2XB5, 1Z1L), for which the maximum-match topology was not ranked among the initial list of 5000 possible topologies. Each of the initial topologies contains one or more wrong matches, in which a correctly predicted secondary structure trace and a correctly predicted sequence segment are matched wrongly. These four cases contain the most complicated secondary structure relationships, with sequence length between 197 and 354 amino acids and 10 to 23 secondary structures. Since the initial topologies do not include a maximum-match topology, contact pair information does not enhance the rank of maximum-match topology. Our current design of enrichment relies on the initial topology.

**Figure 4:**
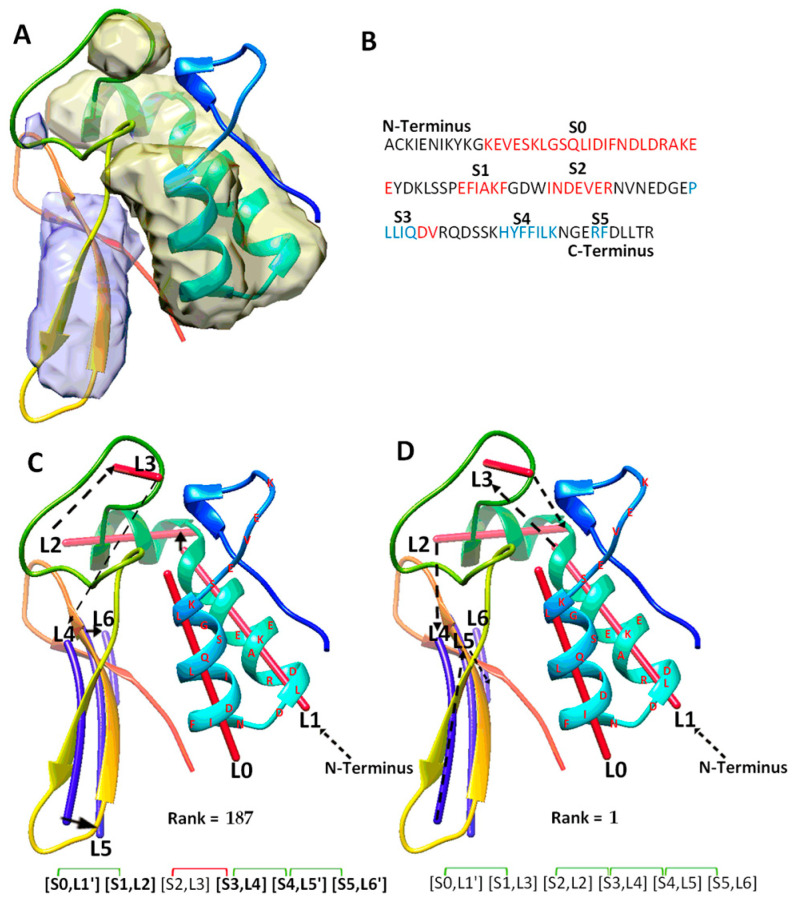
The maximum-match topology for case T1031. (**A**) The atomic structure (rainbow ribbon) is superimposed with the secondary structure regions of α-helices (yellow density) and β-sheet (blue density) detected using DeepSSETracer [20]. (**B**) The amino acid sequence and secondary structures, helices (red) and β-strands (blue), predicted using JPred [30]. (**C**) The 187th ranked initial topology is indicated with black arrows, α-traces (red lines) and β-traces (blue lines) from N to C terminal of the protein sequence. Correctly mapped secondary structure pairs are highlighted in the representation of the maximum-match topology. Secondary structure contact pairs are marked with green and red for satisfaction and dissatisfactory respectively. (**D**) The 1st ranked topology shown similarly as in (**C**).

The fourth category includes a special case (T1031), for which the use of significant long-range pairs worsens the rank of the maximum-match topology. T1031 contains four helices and three β-strands in the atomic structure (Table 2). DeepSSETracer correctly detected three helices (L0, L1, L2), missed a one-turn helix, and wrongly detected a helix (L3) at a turn region. It also detected all three β-strands (L4, L5, L6) (Figure 4C). A main problem with this case is that one and a half of two helices (at L0, L1 region) were predicted as one long helix, S0, using JPred (Figure 4B). The predicted sequence segment merged most of two separate helices into one. The merge mistake in the prediction creates a major problem for matching between the sequence segments and image traces. This is because one of the two correctly detected traces (L0 and L1) is not utilized, and the loop estimation is not accurate between S0 and S1. Additionally, S2 is a wrongly predicted helix at the loop region of the sequence. In spite of the errors in predicted secondary structures from both sequence and the image, the maximum-match topology was ranked 56th on the initial list of topologies. When the constraint pair (S2, S3) was applied to the wrongly predicted S2 helix, violation of constraints shows when the corresponding pair (L3, L4) is measured for distance satisfaction. As a result, the maximum-match topology was re-ranked lower at the 187th. Results from this case suggests that contact pair constraints may hurt the topology ranking when secondary structure predicted is quite wrong, either due to the sequence or the image, as for the case T1031 where two of the three helices predicted are wrong.

## 3. Methods

### 3.1. Preparation of Data

A set of fourteen cases were used to evaluate the effect of using amino acid contact pair information. Each case consists of a sequence and its corresponding density map of the chain. The atomic structure and its amino acid sequence were downloaded from PDB. Although most of the downloaded sequences have atomic structures through the entire length, it is common to see certain portions of the sequence without atomic structures, particularly at the N and C terminals. Those sequence segments that do not have atomic structures were deleted. In details, the deletion was performed in the N terminal for 3948-6esg-B and 2620-4uje-BH, in the C terminal for 6810-5y5x-H, and in the N and C terminals for 9534-5gpn-Ae, 8357-5t4o-L, 3LTJ, 1HG5, 3ACW, and 1Z1L. The original length and the length after deletion are shown in Table 2. By forcing the sequence to be exactly the same as in the atomic structure, it enforces the correspondence between the sequence and the density map in each case, since the density map is produced using the atomic structure. The density maps of six cases are cryo-EM maps downloaded from EMDB (Table 2). Since a cryo-EM density map often consists of multiple chains of proteins and/or nucleotides, components of density map were extracted using the atomic structures of individual chains through Chimera [47]. Since there are no cryo-EM density maps corresponding to 3LTJ, 2XB5, 1HG5, 3ACW, 1Z1L, and the three CASP targets (T1029, T1031, T1033), their density maps were simulated to 8 Å resolution using the atomic structures and the molmap function of Chimera [47]. The skeleton of a density map was derived using SkelEM [48], and it is part of input data for MultiTopoDP. The secondary structure sequence segments were predicted using the sequence that corresponds to its density map, and default parameters were used at the server of JPred [30]. Predicted helices with less than three amino acids are ignored. Secondary structure positions were annotated from the atomic structure using STRIDE [49].

### 3.2. Protein Secondary Structure Contact

Amino acid contact prediction was performed using DNCON2, which is a tool of MULTICOM software [40,41], for the eleven cases (6810-5y5x-H, 9534-5gpn-Ae, 8518-5u8s-A, 3948-6esg-B, 2620-4uje-BH, 8357-5t4o-L, 3LTJ, 2XB5, 1HG5, 3ACW, 1Z1L) and RaptorX [42] for the three targets of CASP(T1029, T1031, T1033) (Table 2). Given an amino acid sequence, DNCON2 produces a list of amino acid contacts, each with a p-value (between 0 and 1). In order to extract significant long-range contacts, screening was conducted to (1) remove all pairs with near zero p-values; (2) remove short-range pairs with less than or equal to 3 amino acids separating them; (3) extract those pairs that have p-values above a threshold. The selection was based on a binned procedure starting with three standard deviations (3SD) of the p-values in a chain, moving to two standard deviations (2SD), and then using one standard deviation (1SD). If 0 or 1 pair of secondary structure contact was obtained using 3SD, 2SD was used as a threshold. If 0 or 1 contact pair was obtained using 2SD, then the threshold was reduced to 1SD. The same screening was performed to amino acids contacts obtained using RaptorX for the three CASP targets. The predicted contact pairs of amino acids were mapped to sequence segments predicted using JPred to identify pairs of secondary structures in contact. The secondary structure contact pairs, the number of significant long-range amino acid contact pairs that are mapped to each pair of secondary structure segments, and the thresholds of p-values are shown in Table 1.

### 3.3. Secondary Structure Traces from Cryo-EM Density Maps

The region of α-helices and β-sheets were detected from the density map using DeepSSETracer, which uses a convolutional neural network to detect secondary structures [20]. DeepSSETracer contains a model that was trained on a set of cryo-EM component density maps at medium resolution. Given the density map in the format of MRC, such as the one in Figure 1A, DeepSSETracer produces two segmented maps, one for detected helices, the other for detected β-sheets. DeepSSETracer can be installed as a bundle to ChimeraX to utilize the visualization capacity of ChimeraX. When two secondary structure regions are connected in the density map, Segger was used to derive individual density regions of secondary structures [50]. For each segmented helix density region, a Python program was written to use Principle Component Analysis (PCA) to derive a line (α-trace) for each segmented helix region. For each segmented β sheet region, StrandTwister was used to predict traces of β-strands [21]. StrandTwister detects the overall orientation of a set of β-strands and generates a small number of alternative sets of β-traces, each differing slightly in the shift and orientation. In this study, the best set that is closest to the true set of β-strands was used.

### 3.4. Generation of Initial Topologies

Secondary structure traces refer to the set of α-traces and β-traces detected from a Cryo-EM density map. The secondary structure sequence segments refer to α-helices or β-strands predicted using existing software such as JPred [30]. MultiTopoDP is a graph-based dynamic programming method to match secondary structure traces with secondary structure sequence segments [17]. The core idea of the method is to translate the matching problem into a 2-dimensional graph problem, in which each node (i,j) represents the assignment of sequence segment $S_{i}$ to trace $T_{j},\;i=1,\;\ldots ,\;M,\;j=1,\;\ldots ,\;N$. An edge weight is used to express the geometrical satisfaction of assigning two neighboring segments on the sequence to two traces in the density map. The problem of matching sequence segments to image traces is then a problem of finding the shortest path under specific constraints. The score of the shortest path is the score for each topology and was used to rank the initial topologies. In addition to the set of traces and the set of sequence segments of secondary structures, MultiTopoDP uses the skeleton of the cryo-EM density map to measure the distance between two traces in 3-dimensional space. The skeleton was derived using SkelEM [48]. Although MultiTopoDP accepts multiple secondary structure predictions as input, only JPred was used in the work of this paper. MultiTopoDP produces a list of top-ranked topologies with scores, as well as the rank of the maximum-match topology.

### 3.5. Re-Rank Topologies Using Secondary Structure Contact Pairs

**Figure 5:**
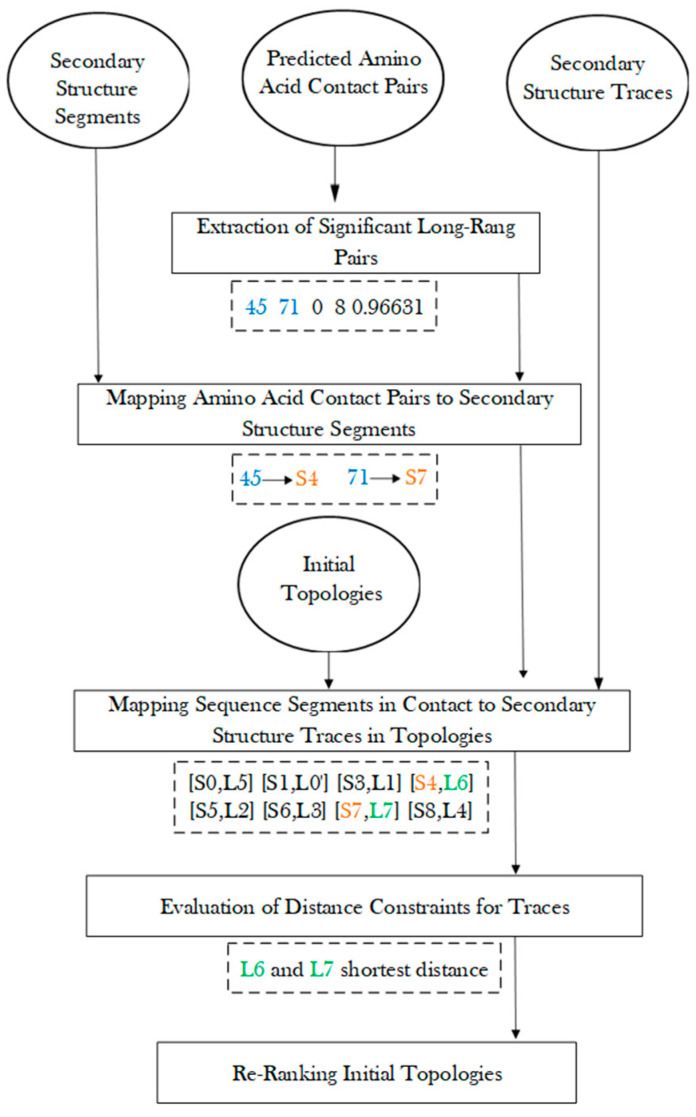
Evaluation of initial topologies using amino acid contact pairs.

After amino acid contact pairs are mapped to secondary structure sequence segments, the secondary structure contact pairs were used to evaluate each initial topology, and those satisfying the contact constraints were ranked higher (Figure 5). For a pair of secondary structure sequence segments that were predicted in contact, their corresponding traces were evaluated for the shortest distance between the two traces. The shortest distance between the pair of traces is defined as the shortest distance between any two points, one from each line. Those pairs of traces with shortest distance less than a threshold of 13 Å were considered as in contact. The threshold was estimated based on the radius of a helix of about 5 Å. Although the threshold of 13 Å is empirical, our exploration of 11 Å and 12 Å as thresholds does not change the overall conclusion. Note that the shortest distance between two line segments is a simple evaluation of contacts between two secondary structures. The actual contact distance often differs slightly depending on where the contact points are located on the line segments. Let *CP* be the number of pairs of secondary structure traces on which amino acid contacts are mapped. Let *SP* be the number of pairs of traces that are predicted to be in contact and actually have shortest distance within 13 Å. The ratio $\frac{SP}{CP}$ was used to sort all initial topologies, and each topology is associated with a such created rank, Rank_update. Let Rank_init be the initial rank obtained using MultiTopoDP for each topology. This reflects how well secondary structure components are matched. The final rank of each topology was obtained by sorting the score of a weighted sum of the initial rank and the updated rank with a weight a = 0.7 (Formula (1)).

(1) score=(a×Rank_init)+((1−a)×Rank_update)

## 4. Conclusions

Since secondary structures are major components of a protein, the knowledge of their relative geometry is important in deriving the tertiary structure of a protein. A topology of secondary structures defines the mapping between a set of sequence segments in 1D and a set of traces of secondary structures in 3D. Although several algorithms have been proposed to derive the topology of secondary structures when a cryo-EM density map is available, it is challenging to obtain high-accuracy topologies due to inaccuracy in both secondary structure predictions and detection of traces from cryo-EM density maps. We developed a framework to rank secondary structure topologies through integration of three sources of information: the secondary structure traces detected from a cryo-EM density map, predicted secondary structure sequence segments, and amino acid contact pairs. Although many aspects can be improved, this is the first work with analysis using three sources of information for the secondary structure topology problem.

The approach was designed to use significant long-range contact pairs to enrich initial topologies generated using MultiTopoDP. A test using 14 cases shows four categories of effect. Enhanced ranking of the maximum-match topology was observed in four cases that contain a large number of secondary structures, and the predicted secondary structures are fairly good from both the sequence and density map. The enrichment is limited in four cases where the initial topology was between 1st and 5th already. It appears that when the maximum-match topology is already highly ranked, additional constraints are needed to distinguish among a small set of top-ranked topologies. Current design depends on the quality of initial topologies. In situations when initial topologies fail to be generated in top-5000-candidate topologies, enrichment also fails. It was observed in one case that when secondary structure prediction is poor, the use of contact pairs could potentially reduce the rank of the maximum-match topology, since contact pairs are mapped wrongly to a pair of traces. Although only significant long-range amino acids pairs are used in this work, the use of more contact information is expected to enhance the effectiveness if a comprehensive screening process can be developed and applied for the measure of constraints in 3D space. Our results show the potential of combining the cryo-EM density maps with well-analyzed contact information in deriving protein structures. Using a simple way to combine three sources of information, the maximum-match topology can be included in a set of top 300 topologies for nine of the fourteen test cases. Our analysis of various cases in details reveals challenges to be overcome in future development.

## Figures and Tables

**Table 1:** Secondary structure contact pairs derived from amino acid contact prediction. Amino acid contact pairs were obtained using MULTICOM [40,41] or RaptorX [42] (see details in Methods). ^a^ The ID of a test case involving a cryo-EM density map is labeled as EMDB ID-PDB ID-chain ID. The ID of a case involving a simulated density map is labeled using either a PDB ID or a CASP target ID. The threshold of p-values (standard deviations (SD)) used for selection of significant long-range pairs is indicated. ^b^ Secondary structure contact pairs are labeled using the IDs of sequence segments predicted using JPred [30], the type of secondary structure indicated as either α or β, and the number of significant long-range amino acid pairs that are mapped on the secondary structure pair.

^a^ Case	^b^ Secondary Structure Contact and Number of Significant Long-Range Pairs
6810-5y5x-H (3SD)	(S4, S5)-(β, α)-1; (S4, S7)-(β, β)-4
9534-5gpn-Ae (1SD)	(S0, S1)-(α, α)-1; (S1, S2)-(α, α)-1; (S2, S3)-(α, α)-3
8518-5u8s-A (3SD)	(S1, S2)-(α, α)-1; (S4, S7)-(β, β)-9; (S4, S9)-(β, β)-3; (S5, S6)-(β, β)-1; (S7, S8)-(β, α)-4; (S8, S9)-(α, β)-1
3948-6esg-B (1SD)	(S0, S1)-(α, α)-3; (S1, S2)-(α, α)-9; (S2, S3)-(α, β)-5
2620-4uje-BH (3SD)	(S2, S3)-(β, β)-22; (S2, S5)-(β, β)-10; (S3,S4)-(β, α)-1; (S4, S5)-(α, β)-1; (S9, S10)-(α, β)-4;
8357-5t4o-L (2SD)	(S0, S5)-(α, α)-2; (S0, S1)-(α, α)-1; (S1, S2)-(α, α)-1; (S6, S8)-(β, β)-24; (S6, S9)-(β, β)-14; (S7, S8)-(α, β)-1; (S9, S10)-(β, β)-5
3LTJ (2SD)	(S3, S5)-(α, α)-1; (S5, S7)-(α, α)-1; (S8, S9)-(α, α)-1
2XB5 (3SD)	(S0, S1)-(α, α)-8; (S0, S3)-(α, α)-2; (S1, S2)-(α, β)-1; (S4, S9)-(α, α)-6; (S4, S6)-(α, α)-1; (S4, S7)-(α, α)-1; (S6, S7)-(α, α)-5; (S7, S9)-(α, α)-3
1HG5 (2SD)	(S1, S3)-(α, α)-5; (S1, S2)-(α, α)-1; (S2, S3)-(α, α)-1; (S3, S4)-(α, α)-2; (S7, S9)-(α, α)-2
3ACW (3SD)	(S2, S6)-(α, α)-1; (S5, S6)-(α, α)-1; (S6, S7)-(α, α)-4; (S7,S10)-(α, α)-1; (S10, S11)-(α,α)-1
1Z1L (3SD)	(S3, S8)-(α, α)-1; (S4, S5)-(α, α)-7; (S5, S9)-(α, α)-7; (S5, S11)-(α, α)-1; (S8, S9)-(α, α)-4; (S9, S11)-(α, α)-3; (S10, S11)-(α,α)-3; (S12, S13)-(α,α)-2; (S3, S4)-(α, α)-1; (S9, S10) (α, α)-2; (S13, S14)-(α,α)-6
T1029 (3SD)	(S1, S6)-(α, α)-4; (S2, S3)-(β, β)-9; (S3, S4)-(β, β)-11; (S4, S5)-(β, β)-12
T1031 (3SD)	(S0, S1)-(α, α)-1; (S2, S3)-(α, β)-1; (S3, S4)-(β, β)-18; (S4, S5)-(β, β)-6
T1033 (3SD)	(S0, S4)-(α, α)-2; (S2, S3)-(α, α)-7; (S3, S4)-(α, α)-6; (S4, S5)-(α, α)-2

**Table 2:** The rank of the maximum-match topology produced using secondary structure sequence segments, traces, and amino acid contact pairs. ^a^ A test case involving a cryo-EM density map is labeled as EMDB ID-PDB ID-chain ID. A case involving a simulated density map is labeled using the PDB ID. A case involving a CASP target is labeled using the target ID. The resolution of a density map is indicated. ^b^ The number of amino acids in the protein (length of downloaded sequence/length in atomic structure). ^c^ The number of α-helices/β-strands in the atomic structure. (+) indicates number of β-strands in each β-sheet. ^d^ The number of α-helices/β-strands predicted using JPred. ^e^ The number of α-traces/β-traces detected from the 3D density map. ^f^ The number of correctly matched secondary structures (α-helices/β-strands) that are included in the maximum-match topology. ^g^ Rank of the maximum-match topology without using contact pairs. ^h^ Rank of the maximum-match topology using contact pairs. *: Merge error in a predicted helix using JPred.

Case ^a^	#a.a.^b^	True Struct. ^c^	Seq Pred. ^d^	Image Detect. ^e^	Max Pairs ^f^	Rank of Maximum-Match Topology	
						No_C ^g^	With_C ^h^
6810-5y5x-H(5 Å)	104/100	5/3	5/4	5/3	6(4/2)	1	1
9534-5gpn-Ae(5.4 Å)	116/88	4/0	4/0	4/0	4(4/0)	2	2
8518-5u8s-A(6.1 Å)	208/208	6/2	5/3 + 2	5/3 + 2	7(5/2)	142	116
3948-6esg-B(5.4 Å)	102/78	3/0	3/1	3/0	3(3/0)	5	5
2620-4uje-BH(6.9 Å)	194/191	7/3 + 3	5/3 + 3	4/3 + 3	10(4/6)	NA	-
8357-5t4o-L(6.9 Å)	177/160	9/0	7/4	8/2	7(7/0)	2	2
3LTJ(8 Å)	201/191	16/0	12/0	12/0	12(12/0)	NA	-
2XB5(8 Å)	207/207	12/0	9/1	10/3	9(9/0)	NA	-
1HG5(8 Å)	289/263	11/0	10/0	13/0	9(9/0)	1022	217
3ACW(8 Å)	293/284	15/0	12/1	12/2	12(12/0)	2072	1141
1Z1L(8 Å)	345/338	23/0	15/0	15/0	13(13/0)	NA	-
T1029(8 Å)	125/125	6/4	3/5	6/4	7(3/4)	437	117
T1031(8 Å)	95/95	4/3	3 */3	4/3	5(2/3)	56	187
T1033(8 Å)	100/100	3/0	6/0	4/0	4(4/0)	5	5

## Data Availability

Data is contained within the article. Please contact the corresponding author for further questions.
